# Contribution and clinical relevance of germline variation to the cancer transcriptome

**DOI:** 10.1186/s12885-022-09757-0

**Published:** 2022-06-20

**Authors:** Bernard Pereira, Emma Labrot, Eric Durand, Joshua M. Korn, Audrey Kauffmann, Catarina D. Campbell

**Affiliations:** 1 Novartis Institutes for Biomedical Research, 250 Massachusetts Avenue, Cambridge, MA 02139 USA; 2 Novartis Institutes for Biomedical Research, Novartis Campus, Fabrikstrasse 2, CH-4056 Basel, Switzerland

**Keywords:** eQTL, TCGA, Cancer genomics, Germline variants

## Abstract

**Background:**

Somatic alterations in the cancer genome, some of which are associated with changes in gene expression, have been characterized in multiple studies across diverse cancer types. However, less is known about germline variants that influence tumor biology by shaping the cancer transcriptome.

**Methods:**

We performed expression quantitative trait loci (eQTL) analyses using multi-dimensional data from The Cancer Genome Atlas to explore the role of germline variation in mediating the cancer transcriptome. After accounting for associations between somatic alterations and gene expression, we determined the contribution of inherited variants to the cancer transcriptome relative to that of somatic variants. Finally, we performed an interaction analysis using estimates of tumor cellularity to identify cell type-restricted eQTLs.

**Results:**

The proportion of genes with at least one eQTL varied between cancer types, ranging between 0.8% in melanoma to 28.5% in thyroid cancer and was correlated more strongly with intratumor heterogeneity than with somatic alteration rates. Although contributions to variance in gene expression was low for most genes, some eQTLs accounted for more than 30% of expression of proximal genes. We identified cell type-restricted eQTLs in genes known to be cancer drivers including LPP and EZH2 that were associated with disease-specific mortality in TCGA but not associated with disease risk in published GWAS. Together, our results highlight the need to consider germline variation in interpreting cancer biology beyond risk prediction.

**Supplementary Information:**

The online version contains supplementary material available at 10.1186/s12885-022-09757-0.

## Background

Deregulated gene expression is a defining feature of the cancer cell and often results in the disruption of key signaling pathways that control cellular growth and proliferation [1]. Analyses of multidimensional data from large-scale projects such as The Cancer Genome Atlas (TCGA) have demonstrated important associations between somatic genetic alterations and changes in gene expression, some of which represent oncogenic alterations in cancer driver genes [2, 3].

In contrast, the role of inherited polymorphisms in influencing the cancer transcriptome has been less well studied. Identification of associations between germline single nucleotide polymorphisms (SNP) and gene expression has been established as a strategy to understand the mechanisms by which inherited variants may influence defined phenotypes, including cancer incidence [4]. However, there have been few studies addressing the biological and clinical relevance of eQTLs in the specific context of the malignant transcriptome.

Tumor biopsies consist of numerous cell populations including immune and stromal cells. As a result, eQTLs identified in tumor samples may be derived from either malignant or non-malignant cell types. This was demonstrated in an analysis of 24 cancer types in which eQTL mapping revealed that genes in which an eQTL was detected (eGenes) were enriched for ontology terms related to immune function [5]. Approaches to identify cell type-restricted eQTLs include adapting the standard additive eQTL model by considering how an eQTL’s effect varies by the estimated fraction of a given cell type in a tissue biopsy [6].

Application of this approach to breast cancer datasets using tumor cellularity as an estimate of tumor cell fraction revealed that only a few of the eQTLs detected in these studies were likely to be tumor cell-specific [7]. More recently, analyses using in silico deconvolution methods have revealed the presence of cell type-restricted eQTLs in at least 3000 genes in the Genotype-Tissue Expression (GTEx) project [8].

In this study, we explored the role of inherited variants in the cancer transcriptome using genotype, somatic alteration and gene expression data from 24 cancer types from the TCGA project. We studied the tissue specificity of eQTLs in cancer, with a focus on characterizing putative functional enrichment in genes with at least one proximal eQTL (eGenes). We quantified the contribution of eQTLs to variation in gene expression relative to somatic alterations and determined that the role and potential relevance of cancer eQTLs likely varies by cancer type. In particular, thyroid carcinoma and prostate adenocarcinoma had the highest number of detected eQTLs across the study, likely driven by the low somatic alteration rates and low levels of intratumor heterogeneity within these cancer types. We also identified cell type-restricted eQTLs that are associated with patient prognosis, demonstrating the potential impact of inherited variants to mediating tumor biology and clinical outcome.

## Methods

### Data sources

We downloaded genotype, mutation and expression datasets from the TCGA from the Genomics Data Commons [4, 9, 10] (GDC). Data stored on the GDC have been processed using standardized pipelines with the hg38 reference genome build. We obtained the clinical data for subjects with samples in TCGA that had already been curated as previously described [11]. Raw genotype data were downloaded on November 28, 2017 and the version of the RNA-seq data used for the final analyses were accessed on October 18, 2019. All somatic alteration data used for the study were obtained on March 13, 2019.

### Genotype processing

We processed genotype data previously run through BIRDSEED [12] and used PLINK [13] to remove low-quality SNPs and outlier samples. First, we remapped probe locations to hg38 using the remapping files provided by the GDC. We then removed genotype calls with BIRDSEED quality scores greater than 0.05. In addition, we removed SNPs with MAF < 0.01 and those with missing calls in more than 95% samples across the entire TCGA cohort from the analysis. We also filtered out individuals with more than 2.5% SNP calls missing. To account for technical artifacts including population stratification in the eQTL model, we performed principal component analysis (PCA) with PLINK to identify potential genetic covariates. By comparing TCGA genotypes with genotypes from Phase 3 of the 1000 Genomes project [14], we observed that the first five principal components were related to ancestry and accounted for 94.4% of the variance in genotypes (Supplementary Fig. 1). Thus, we included 5 principal components in our eQTL model.

### Genotype phasing and imputation

We first phased genotypes with Eagle 2 [15], using data from Phase 3 of the 1000 Genomes project as a reference. All 1000 Genomes Project SNP coordinates were lifted over to reference genome hg38 prior to phasing. We performed phasing for all samples in the TCGA dataset together, and then separated samples by cancer type. We then imputed genotypes across the genome using Minimac 4 [16] and SNP calls from 1000 Genomes data with default settings. Finally, we filtered SNPs with MAF < 0.01 and R-squared less than 0.3, as recommended in the Minimac 4 guidelines. The final dataset consisted of approximately 10–11 million SNPs per sample.

### RNA-seq data processing

We obtained raw HT-seq [17, 18] counts and RNA FPKMs for all samples from the GDC, which were generated with standardized alignment and read counting pipelines. We normalized data using the geometric mean method in DESeq2 [19] and further transformed the normalized counts using DESeq2’s variance stabilizing transformation for QC assessment and visualization. We performed PCA using the transformed data to identify sample outliers and removed individuals whose expression profiles lay more than three standard deviations away from the mean on any one of the six first principal components. In addition, we filtered lowly expressed genes, retaining only those with at least 0.1TPM and 6 reads in a minimum of 20% of the samples for each cancer type. These thresholds are similar to those used for GTEx analyses. To identify expression covariates for inclusion in the eQTL model, we used PEER [20], and included the first 15 PEER factors for each cancer type in the subsequent analyses.

### eQTL mapping

We normalized expression data using a rank-based inverse normal transformation as described for GTEx [21]. We used a two-part regression for eQTL mapping, first regressing gene expression on genotype PCs, PEER factors, patient sex, copy number-changes and inactivating point mutations for each gene. We focused only on high-level amplifications and deep deletions and coded these + 1 and -1 respectively. We then normalized residuals from this regression using the rank-based inversed normal transformation, and used the resulting gene expression values as input to FastQTL [22]. We restricted analyses to variants for which the variant allele was observed at least five times in each cancer and then ran FastQTL with the parameters ‘—permute = 1000,10,000, –window = 1e6’. Mapping was performed using the imputed genotype dosages. The resulting p-values were corrected using Storey’s q-value method, and associations with FDR = 0.05 were retained. We used the same multiple testing correction strategy for somatic copy number alterations (CNAs) and inactivating mutations. We randomly sampled 200 patients from each cohort for downsampling analyses.

### eQTL downstream analyses

We used the package variancePartition [23] to deconvolute gene expression variance into the relevant technical and genetic factors within each cancer type. In general, we deconvoluted variance for all covariates included in the additive eQTL model except for cases where data was missing or there were no somatic alterations in a gene. To evaluate the effects of individual variants, we used the allelic fold change method that has previously been described [24]. Functional enrichment of GO terms was analyzed using the R package goSeq [25], and the resulting p-values were corrected for using the Benjamini–Hochberg method. We defined a similarity index for two cancer types as the intersection divided by the union of the detected eGenes. This index was scaled between 0–1 for the purpose of visualization and interpretation. To assign gene type and gene boundaries, we used gene annotations from GENCODE v.22 (hg38), which was also used by the GDC for counting RNA-seq reads. We obtained ABSOLUTE-derived copy number instability estimates, genomic purity estimates and CIBERSORT [26] cellular fractions from previously published analyses [27]. We used the Consensus Purity Estimates (CPE) previously computed [28–30] as measurements of tumor purity, which considers IHC, gene expression, methylation and copy number data. In addition, we computed a version of the previously defined mutant allele tumor heterogeneity (MATH) statistic [31] to summarize intratumor heterogeneity for each sample. We calculated cancer cell fractions (CCF) [32] for all mutations in the dataset and defined the MATH score as the median absolute deviation in CCF divided by the median CCF for each patient.

### Interaction and survival models

Following previously described strategies [33] we built cell type-restricted models by including an interaction term between genotype and tumor purity (expression = genotype + technical covariates + somatic alterations + purity + genotype*purity). We used a modified version of FastQTL to perform interaction analyses (https://github.com/francois-a/fastqt)l. and an FDR = 0.1 for identifying significant interactions thus accounting for the low power of detection in the interaction model. Where appropriate, we performed survival analyses using Cox proportional hazards models accounting for covariates as described in the text.

## Results

### The eQTL landscape in human cancers

**Fig. 1:**
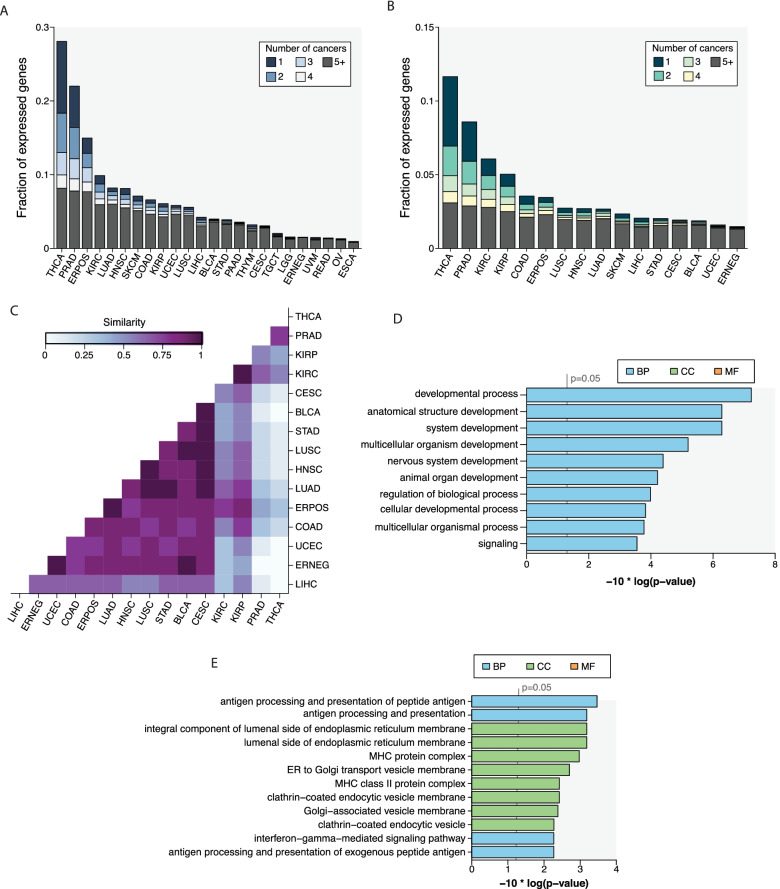
The eQTL landscape in 24 cancer types. **A** The proportions of expressed genes with evidence of germline regulation are shown for 24 cancer types from the TCGA. The number of cancer types in which eGenes are found are indicated by the colored bars. **B** eGenes identified after randomly selecting 200 patients from each cancer cohort. As before, bar colors represent tissue sharing between eGenes. Only cancers for which at least 200 patients were available are shown. **C** Similarities in eGene profiles between cancer types after downsampling. The similarity index was derived by considering the ratio of the intersection to the union of eGenes present in all pairs of cancers. On the adjusted scale, 0 = the lowest and 1 = the highest similarity observed between two different cancer types. **D** Overrepresentation analysis of GO enrichment terms when considering only cancer type-specific eGenes among all ubiquitously eGenes, **E**  Overrepresentation analysis of GO enrichemtn terms when considering shared eGenes (present in at least 10 cancer types) among all ubiquitously expressed eGenes. Enrichment analyses were performed considering all three GO categories (*CC *cellular compartment; *BP* biological process, *MF* molecular function) using goSeq. Adjusted *p*-values are shown for ten most significant GO terms

We mapped proximal associations (within 1 Mb of gene boundaries as defined in GENCODE v. 22) between common polymorphisms (minor allele frequency; MAF > 1%) and gene expression in each of 24 cancer types from the TCGA project (estrogen receptor-positive (ER +) and ER- breast cancers were considered separately) (Table 1). In each cancer type, we employed an additive model that accounted for high-level somatic copy number events and inactivating point mutations (Methods). Overall, we identified 8,857 eGenes in at least one cancer type at a 5% genome-wide FDR, including 54 eGenes shared between all cancers and 4,346 eGenes present in only one cancer type (Supplementary Fig. 2A). The proportion of expressed genes **(**Supplementary Fig. 2B**)** that were also eGenes ranged between 0.9% (esophageal cancer) – 28.1% (thyroid cancer; median = 4.1%) with the highest proportion of eGenes/expressed genes in thyroid, prostate (22.0%), and ER + breast (15.0%) cancers (Fig. 1A). As the power to identify an eQTL is related to the number of patients in an indication (Spearman’s rho between sample size and eGene/expressed gene fraction = 0.83, *p* < 0.001; Supplementary Fig. 3**)**, we repeated the analysis after downsampling each cancer type to 200 patients. In the 16 cancer datasets with at least 200 patients available, we identified eGenes in between 1.5% (ER- breast cancer) – 11.7% (thyroid cancer; median = 2.7%) of expressed genes using the downsampled data (Fig. 1B). Interestingly, previous eQTL analyses in normal tissue have also identified high number of eGenes in thyroid and prostate tissues [34, 35]. When using the downsampled data, there were 71 eGenes shared between all 16 cancer types and 2,184 eGenes unique to a single cancer type. Of these 2,184 unique eGenes, 1,572 (72.0%) were expressed in all 16 cancer types. This suggests that even genes that are expressed in multiple cancer types may be regulated by germline variants in only a subset of cancers.

**Table 1:** TCGA cohorts used in the study. Patient numbers include those who passed filtering criteria and had complete datasets (somatic alteration, gene expression, genotyping data) available. Only cancer types with at least 200 patients were used in downsampling analyses

Code	Cancer types	Number of patients	Included in downsampling
BLCA	Bladder carcinoma	320	Y
ERPOS	ER + breast carcinoma	659	Y
ERNEG	ER- breast carcinoma	203	Y
CESC	Cervical squamous cell carcinoma	246	Y
COAD	Colorectal adenocarcinoma	334	Y
ESCA	Esophageal carcinoma	143	N
HNSC	Head and neck squamous cell carcinoma	438	Y
KIRC	Kidney renal clear cell carcinoma	279	Y
KIRP	Kidney renal papillary cell carcinoma	230	Y
LGG	Low-grade glioma	88	N
LIHC	Liver hepatocellular carcinoma	308	Y
LUAD	Lung adenocarcinoma	443	Y
LUSC	Lung squamous cell carcinoma	355	Y
OV	Ovarian serous cystadenocarcinoma	168	N
PAAD	Pancreatic adenocarcinoma	139	N
PRAD	Prostate adenocarcinoma	443	Y
READ	Rectal adenocarcinoma	112	N
STAD	Stomach adenocarcinoma	293	Y
SKCM	Skin cutaneous melanoma	433	Y
TGCT	Testicular germ cell tumors	122	N
THCA	Thyroid carcinoma	448	Y
THYM	Thymoma	113	N
UCEC	Uterine corpus endometrial carcinoma	458	Y
UVM	Uveal melanoma	78	N

### eGene sharing between cancers

To identify similarities in eQTL profiles between cancers, we computed Jaccard similarity coefficients based on the overlap in eGenes between each pair of cancer types in the downsampled data **(**Fig. 1C; Methods). The coefficients ranged between 0.10 (thyroid and ER- breast cancer) – 0.43 (lung squamous cell carcinoma and lung adenomacarcinoma).

To compare relative similarities, we scaled the coefficients between 0–1, where 1 represented the highest similarity observed between two different cancers. As expected, given the relatively high proportion of unique eGenes in this cancer type, thyroid cancer was, on average, most dissimilar from the other cancer types. Nevertheless, thyroid cancer was most similar to prostate adenocarcinoma (scaled similarity coefficient = 0.73) and kidney renal cell cancer (scaled similarity coefficient = 0.58), supporting the notion that these three cancer types have substantially different eGene landscapes. Cancers with common histologies including lung adenocarcinoma and lung squamous cell cancer (scaled similarity coefficient = 0.94), and kidney renal cell cancer and kidney renal papilloma (scaled similarity coefficient = 0.95) also had similar eGene profiles. Interestingly, there was a lower overlap between ER + and ER- breast cancer (scaled similarity coefficient = 0.83), and ER- breast cancer was more similar in eGene composition to uterine cancer (0.98), bladder cancer (0.92) and cervical cancer (0.9). This may reflect similarities in somatic alteration profiles at the genomic level: all three cancer types, for example, are characterized by frequent mutation of TP53 and basal cell-like phenotypes. Together, these results demonstrate that although the overall eGene distribution resembles that found in normal tissues, differences in eGene profiles are sometimes apparent, potentially due to variation in tumor biology.

Next, to better understand the relevance of genes whose expression is under genetic control, we characterized the functional relevance of eGene sharing by performing Gene Ontology (GO) enrichment analysis. We first analyzed the 17,827 ubiquitously expressed genes to generate a background model. When comparing to this background set of genes, there was an overall enrichment for terms related to immune and MHC function, and metabolism within the 3,696 eGenes identified using downsampled data **(**Supplementary Fig. 4A). The eGene enrichment for immune function is consistent with the presence of eQTLs in infiltrating immune cells in heterogeneous tumor biopsies, and has been previously described in the cancer setting [36]. In the remaining 14,131 non-eGenes, there was significant overrepresentation of terms related to RNA biosynthesis and transcription (Supplementary Fig. 4B**)** indicating that the expression levels of genes involved in biosynthesis are less subject to germline regulation. As we were specifically interested in better understanding eGene sharing between cancers, we repeated the enrichment analysis this time using only the 3,696 eGenes in the background model. Comparison of unique eGenes (present in only one cancer type) with all identified eGenes included overrepresentation of functional terms related to developmental processes, although we did not find any statistically significant enrichment when considering these unique eGenes alongside the entire set of 17,287 genes in the background model (Fig. 1D). On the other hand, terms related to immune function were enriched for among shared eGenes (defined as eGenes detected in at least 10 cancer types) (Fig. 1E). Given the large number of thyroid cancer-specific eGenes detected and the intersection between developmental and cancer pathways, this result suggests that germline variation may be especially relevant for cancer biology in thyroid cancer and should therefore be considered alongside the spectrum of somatic alterations in this cancer type.

### Contribution of eQTLs to the cancer transcriptome

**Fig. 2:**
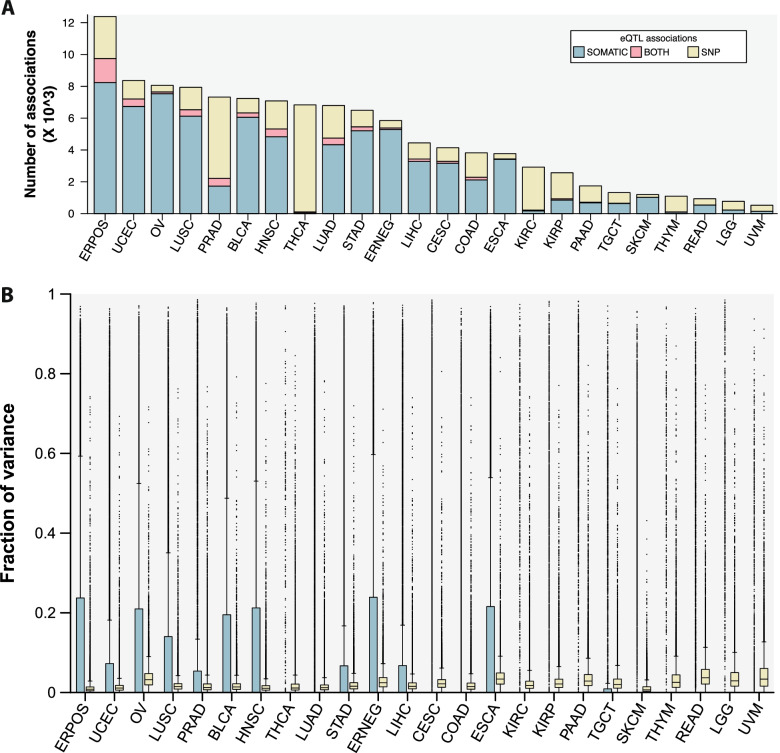
Contributions of eQTLs to the cancer transcriptome**.** (A) Numbers of total variant-expression associations identified across all cancer, where variants refer to inherited SNPs, somatic point mutations and somatic copy number changes. The proportions of these different classes of variants are also indicated. *SNP*  germline variants, *SOMATIC*  somatic variants, *BOTH*  germline and somatic variants**.** (B) Fraction of variance of individual genes attributable to different genetic factors. Gene experience variance was deconvoluted into all technical and biological factors included in the eQTL model (Methods) and the results for the genetic factors are shown here. Colors correspond to those in (A)

Having characterized the global landscape of eQTLs in tumors, we next sought to understand the relative contribution of eQTLs to the cancer transcriptome and differences between cancer types. We first assessed the number of eQTLs and significant somatic variant-expression associations (somQTLs; high-level copy number amplifications, deep deletions, inactivating point mutations), as determined using the additive model, for all samples within each cancer type. The total number of variant-expression associations varied by the number of patients available for each cancer type, ranging between 527 (uveal melanoma) – 12,388 (ER + breast cancer; median = 4294) (Fig. 2A; Supplementary Fig. 5A). eQTL/somQTL ratios varied between 0.07 (ovarian cancer) – 62.3 (thyroid cancer; median = 0.47) (Supplementary Fig. 5B). After downsampling, the ratios of eQTL/somQTL were similar to the ratios observed in the complete dataset (Supplementary Fig. 5C). This reflects the fact that the relative power to detect eQTLs and somQTLs across cancer types is similar given that both eQTL and somQTL analyses are performed using the same datasets within each cancer type, and we subsequently used the complete datasets for our following analyses.

We next used a linear mixed-effects model [5] to quantify the relative contributions of genetic factors to the variance in expression for each gene (Fig. 2B). Overall, median fractions of the proportion of variance attributable to either inherited variants or somatic alterations in a single gene were low. Nevertheless, somatic alterations accounted for the greatest proportion of variance in expression on a per-gene basis across all cancer types (mean proportions of variance for somQTLs = 0.02 (thyroid cancer) – 0.15 (ER + breast cancer); mean proportions of variance in expression accounted for by eQTLs were lower (0.01 (skin cutaneous melanoma) – 0.05 (uveal melanoma)). For example, in ER + breast cancer, somatic alterations accounted for at least 50% of variance in the expression of 4,158 genes but germline eQTLs accounted for at least 50% of variance in the expression of only 24 genes. Across all cancer types, germline variants accounted for at least 30% of expression variance in 522 genes, with 243/522 (46.6%) of these high-variance eGenes present in a single cancer type. In addition, 21 of these genes were eGenes almost universally across cancer types including five putative long non-coding RNAs, genes known to play a role in the immune response [24] (ICOSLG, ERAP2) and U2AF1, which is involved in spliceosome function. Our results indicate that somatic alterations dominate the cancer transcriptome, but that eQTLs can be important mediators of expression of some genes.

Finally, to understand the eQTL landscape in tumors relative to normal tissue, we compared eQTLs identified in 94 paired breast tumor and normal samples from the TCGA as breast was the indication with sufficient normal samples to facilitate a comparison. In the tumor samples, we identified 45 eGenes, of which 23/45 (51%) were unique to tumors. We identified 50 eGenes in the normal samples, including 28/50 (56.0%) eGenes detected only in normal samples.

### Factors contributing to eGene detection

**Fig. 3:**
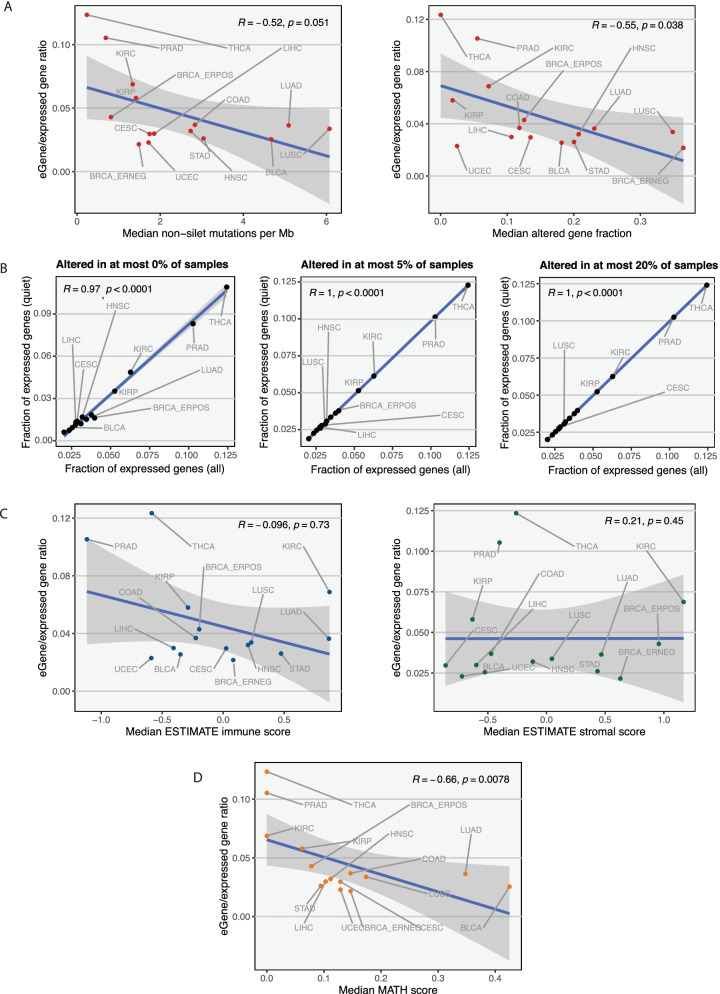
Detection of eQTLs in cancer tissue**.** **A** Correlation between somatic alteration rates and eGene fractions in the downsampled dataset. (Left) Correlation between tumor mutation burden and eGene fraction. (Right) Copy number alteration rate was measured by the fraction of genome altered by any type of copy number alteration. Spearman’s correlation statistics are shown. **B** Correlation between eGene fraction when considering all genes (x-axis) and when considering quiet genes only (those altered in subsets of samples) in the downsampled dataset. (Left) Quiet genes defined as those with no somatic alteration in any sample. (Middle) Quiet genes defined as those with somatic alterations in at most 5% of samples. (Right) Quiet genes defined as those with somatic alterations in at most 20% of samples. Only genes expressed in all samples were used in this analysis. Spearman’s correlation statistics are shown. **C** Correlations between tumor microenvironment scores as defined by ESTIMATE and eGene fraction. Standardized immune (left) and stromal (right) scores defined by ESTIMATE were obtained for all cancer types using the downsampled data, and the median score is shown for each cancer type. Spearman’s correlation statistics are shown. **D** Correlation between levels of intratumor heterogeneity and eGene fraction in downsampled data. The mutant allele tumor heterogeneity (MATH) score was computed for every sample (Methods) and the median MATH score was used to summarize intratumor heterogeneity for each cancer type. Higher MATH scores indicate higher levels of intratumor heterogeneity. Spearman’s correlation statistics are shown

Given the large differences in the number of eGenes across indications, we examined additional genomic data to test for factors that may explain this difference **(**Supplementary Fig. 6). We first explored correlations between somatic alteration rates and eGene fractions in the downsampled datasets (Fig. 3A). The analysis revealed that indications with higher numbers of eGenes had lower somatic alteration rates than other cancer types in general. To further explore whether the effects of somatic alterations on gene expression was impacting our power to detect eQTLs, we focused on ‘quiet’ genes that are infrequently altered by copy number changes or point mutations (Fig. 3B). We observed that, even when removing genes with high somatic alteration rates from the analysis, cancer-specific patterns of eGene/expressed gene fractions remained similar to those from the dataset with all genes. This observation held true when defining quiet genes using different thresholds (genes with no somatic alterations, genes altered in no more than 5% of samples and genes altered in no more than 20% of samples). This result suggests that somatic alteration rates are not solely responsible for the differing numbers of detected eGenes across cancer types.

We next considered whether cellular heterogeneity within a biopsy influenced eQTL detection as the effect of an eQTL in a single cell type may be diluted in the presence of numerous other cell types. We first explored associations between stromal and immune infiltrate in a biopsy and eGene fraction in cancer using previously published estimates of immune and stromal cell tumor infiltration [5] (Fig. 3C; Supplementary Fig. 7). Although there were no significant correlations between eGene fraction and levels of either cell subpopulation, we observed that prostate adenocarcinoma and thyroid cancer had the lowest levels of immune infiltration across all cancer types. Expanding on this observation, we next reasoned that cellular diversity in cancer may also be mediated by varying levels of intratumor heterogeneity, which reflect the number of subclones present within a tumor biopsy. We adapted the previously published MATH score to measure global intratumor heterogeneity (Methods) [33] and observed that eGene fraction was positively correlated with the median MATH score for each cancer type (Fig. 3D). As higher MATH scores indicate higher levels of intratumor heterogeneity (i.e. the presence of more subclones within a tumor), this result suggests that more eGenes were detected in tumors with lower levels of intratumor heterogeneity. Thus, the strongest predictors of number of eGenes in a particular cancer type appear to be tumor intrinsic (subclonality) although we cannot rule out an additional role for tumor extrinsic factors in some cancer types.

### Using an interaction model to identify cell type-restricted eQTLs

**Fig. 4:**
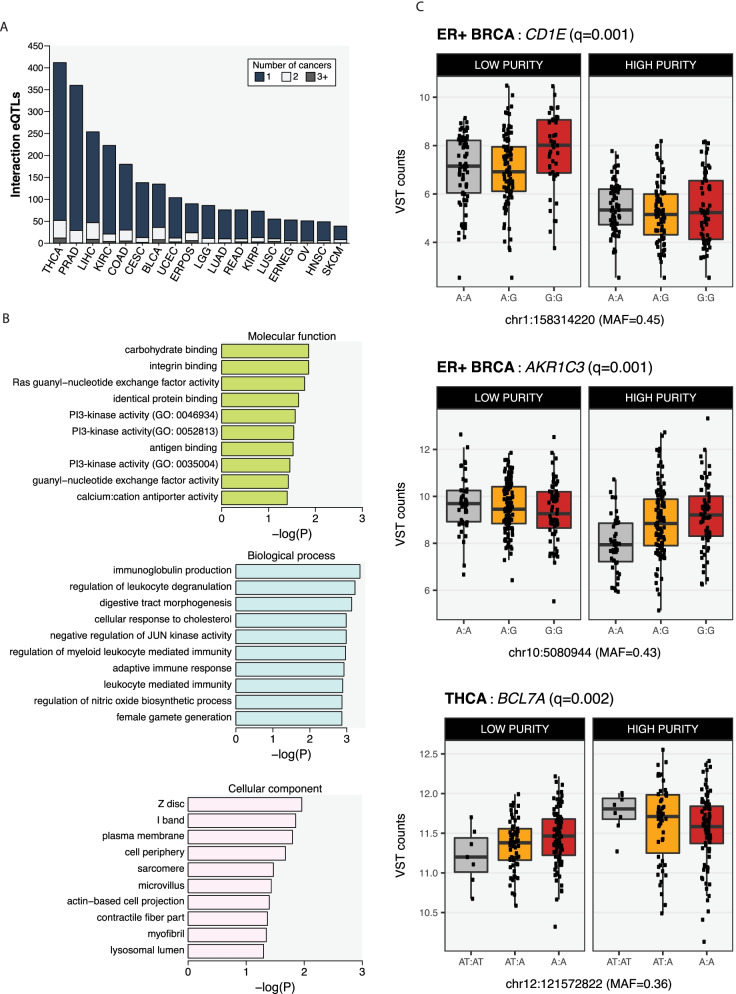
Detection of interaction eQTLs in heterogeneous biopsies. An interaction term was added to the eQTL model to identify ieQTLs, which are likely to be cell-type specific. **A** Total number of ieGenes identified using all patients for each cancer type. As for Fig. 1, the degree of ieGene sharing is also indicated. **B** GO enrichment terms related to the ieQTLs. The three GO categories were considered separately. **C** ieQTL associations in high and low-purity tumors as defined by the upper and lower tertiles of the Consensus Purity Estimate score (Methods). Germline variants are associated with CD1E expression in only low-purity but not in high-purity ER + breast tumors (upper panel), but are associated with AKR1C3 expression in high but not in low-purity ER + breast tumors (middle panel). In thyroid cancer, an ieQTL is associated with BCL7A expression in both high and low-purity tumors but in opposite directions (lower panel). Q-values indicate the adjusted *p*-values (Methods) associated with the interaction term in the eQTL model

Given the association of cell-type heterogeneity with eQTL identification, we sought identify eQTLs specific to tumor cells or specific to the tumor microenvironment. We repeated our analyses, this time including an interaction term between genotype and tumor purity (Supplementary Fig. 6C) in the additive model. This updated model allowed us to detect eQTLs whose effects varied by tumor purity, allowing us to infer cell-type restricted eQTLs in heterogeneous tissue biopsies, although the exact cell types in which the eQTL is acting cannot be determined [31]. Using this approach in the entire cohorts for each cancer, we identified 2,271 interaction eGenes (ieGenes) at FDR = 10%, across all cancer types (Fig. 4A). These included 412 ieGenes in thyroid cancer and 360 ieGenes in prostate adenocarcinoma. A high number of ieGenes was also found in liver cancer (254) despite the relatively small number of eGenes identified in this cancer type using the base model. ieGenes are largely indication-specific with only 160 of the 2,271 (7%) ieGenes shared across indications and only 21 of 2,271 (0.9%) shared between more than 2 cancers. There was no association between the number of ieGenes observed and the median purity estimate obtained for a cancer type.

We next sought to identify the potential functional roles of ieGenes using GO enrichment analysis to study overrepresentation of functional terms (Fig. 4B). Terms related to immune function, stromal cells and cell adhesion were overrepresented over the full set of ieGenes, emphasizing that modelling expression based on tumor purity allows for identification of ieGenes in the non-tumor cells within a biopsy. Nevertheless, we did observe an enrichment of Molecular Function terms related to signaling and growth, mainly through modulation of the phosphatidylinositol 3-kinase pathway, which may be from tumor-restricted eQTL.

We hypothesized that we may be able to differentiate between tumor-specific and other ieQTLs based on the effects observed in when considering ‘high-purity’ and ‘low-purity’ tumors separately. We separated tumors based on whether their associated purity estimates were within the upper or low purity tertile for that cancer type. This allowed us to identify three classes of ieQTLs: those that were associated with gene expression in low-purity tumors but not in high-purity tumors, those that were associated with gene expression in high-purity tumors but not in low-purity tumors, and those were associated with gene expression in high- and low-purity tumors but in opposite directions (Fig. 4C). For example, an ieQTL was associated with CD1E expression in ER + breast cancer only in low-purity tumors. The CD1E gene encodes a protein involved in lipid antigen presentation and was expressed at higher levels in low-purity tumors suggesting that the gene is likely to be expressed in non-tumor cells. In contrast, an association between an ieQTL and AKR1C3 expression was detected in only high-purity tumors suggesting that this ieQTL may be tumor-restricted. Finally, germline variants in BCL7A had opposite effects in thyroid cancer when stratifying tumors by purity. In low-purity thyroid tumors, the variant ieQTL allele was associated with slightly lower BCL7A expression, but the same allele was associated with higher BCL7A expression in high-purity thyroid tumors. This observation suggests that the ieQTL function in BCL7A may differ between tumor and non-tumor cells within the context of thyroid cancer. These examples demonstrate that cell type-restricted eQTLs can be detected in tumor biopsies and that it may be possible to infer whether these act within or outside the tumor compartment.

### Tumor-specific eGenes and patient outcome

**Fig. 5:**
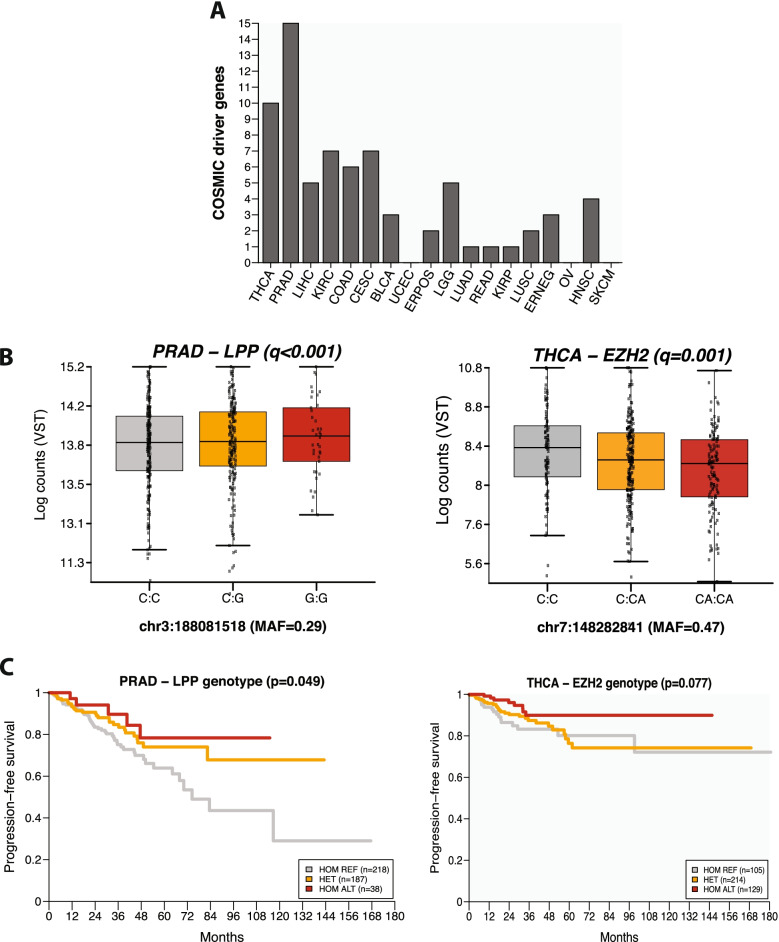
Clinical relevance of interaction eQTLs. **A** ieGenes that were also identified in the COSMIC Cancer Gene Census. **B** Associations between genotypes in LPP and EZH2 and gene expression across all samples in PRAD and THCA. As in Fig. 4, q-values indicate the adjusted p-values associated with the interaction terms in the expanded model. **C** Kaplan–Meier curves showing the association between SNP genotypes and progression-free survival. Hazard ratios were computed when considering genotypes in an additive model

Based on our observations that AKR1C3 and BCL7A have known functions in cancer and that ieQTLs in these genes are likely to be specific to tumor cells, we looked for other ieGenes across the datasets that may also play a role in cancer biology. Using the curated driver gene list from the COSMIC Cancer Gene Census [37]; genes are included on this list based on the presence of likely oncogenic somatic genetic variants. We identified 68 cancer-driver ieGenes (Fig. 5A). These included ABL1 and the master regulator NKX2-1 in thyroid cancer, ERBB3 in liver cancer and AKT3 in colorectal adenocarcinoma. Interestingly, expression levels of the ieGenes LPP in prostate adenocarcinoma and EZH2 in thyroid cancer have previously been associated with patient prognosis in cancer [38], and we observed a similar association within these two cancer types in the TCGA dataset (Supplementary Fig. 8).

Given the associations between patient genotype and gene expression for both LPP and EZH2 (Fig. 5B), we looked for associations between genotype and progression free- survival for these two genes. Our analysis revealed that the genotypes of the lead ieQTLs for both these genes were associated with patient outcomes (Fig. 5C). In thyroid cancer, the alternate allele for EZH2 was associated with lower expression of the gene and consequently longer progression-free survival (hazard ratio, HR, from univariate Cox proportional hazards model = 0.68; 95% confidence interval, CI = 0.47–1.00) consistent with the oncogenic role of EZH2 in cancer. Similarly, the alternate allele for LPP in prostate adenocarcinoma was associated with higher expression of the gene and better outcome (HR = 0.65, CI = 0.45–0.93). To test whether these observations were influenced by residual patient ancestry effects, we repeated the analysis focusing only on patients of European ancestry. In 301/448 (67.2%) thyroid cancer patients of European ancestry, the hazard ratio associated with the EZH2 eQTL modelled as an additive term was 0.64 (CI = 0.40 – 1.02), whereas the LPP eQTL was associated with a hazard ratio of 0.62 (CI = 0.45–0.93) in 356/443 (80.4%) prostate adenocarcinoma patients with European ancestry. These observations highlight the potential of germline variants acting through eQTLs to influence patient clinical trajectories.

## Discussion

In this study, we have characterized the landscape of eQTLs in cancer, focusing on tumor-specific factors that may contribute to detection of eGenes, tissue specificity of eGenes and the possible roles ieGenes may play in mediating tumor biology and patient outcome.

We note that the overall landscape of eGenes across the cancer types reflects the landscape found in normal/non-cancerous tissues for GTEx, with the highest number of eGenes found in thyroid and prostate in GTEx, and in thyroid cancer and prostate adenocarcinoma in our analyses. Given the differences in sample numbers, tissue types and covariates used in the eQTL models, it is not possible to robustly compare eGene detection between the two datasets. While there were also insufficient samples in the TCGA dataset to perform a comprehensive analysis of eGenes in matched tumor/normal pairs, we observed differences in the detected eGenes between breast tumor and normal samples. Overall, we expect some differences in eQTL detection between tumor and normal samples to be due to the presence of somatic alterations in most cancers that dominate gene expression variance in the cancer transcriptome. Consequently, underlying tissue-specific regulatory mechanisms remain similar between normal and malignant tissue, but that there may be eQTLs that are specific to the cancer disease-state and future studies with larger numbers of paired tumor and normal samples would be needed to investigate the role of such tumor-specific eQTLs. The observation that gene expression variance attributable to somatic variants is generally higher than that attributable to germline genetic variants is in contrast to a previous study [5]. This discrepancy is most likely due to differences in coding copy number state in the additive models used in eQTL detection. We note that other strategies for modelling somatic copy number alterations may also influence eQTL detection and that the magnitude of change in expression is likely to be different between deep deletions and high-level amplifications. Factors contributing to eGene discovery both in GTEx and in TCGA include the cellular diversity present within a tissue sample, which most likely varies in an organ- or indication-specific manner [5]. In addition, the possible associations between eGene detection and cancer-specific features such as tumor mutation burden (TMB), chromosomal instability (CIN) and intratumor heterogeneity suggest additional factors that are relevant for eQTL detection in the malignant context. In particular, the association of higher levels of intratumor (sub-clonal) heterogeneity with lower numbers of eQTLs points to the complexity of the cancer transcriptome and the possibility of sub-clonal eQTLs, which would be difficult to identify in bulk gene expression data.

As has been described for normal tissues, genes without an eQTL (non-eGenes) are enriched for genes whose functions are related to cellular development. In tumors, non-eGenes were also enriched for those involved in transcription and RNA metabolism. We expect that the relative depletion of transcription-associated terms among cancer eGenes reflects increased transcriptional activity in cancer cells that are driven either directly or indirectly by somatic alterations and subsequently changes in pathway activity. These changes may disrupt the existing germline regulation of transcription in normal cells. Nevertheless, we observe significant overrepresentation of GO terms related to development when considering cancer type-specific eGenes relative to shared eGenes, which are enriched for terms related to immune and stromal function.

Previous studies have used eQTL analyses in the context of normal tissue to map the biological mechanisms by which SNPs identified through GWAS increase risk of cancer onset [39]. In contrast, the role of eQTLs in the context of the malignant transcriptome and the effects of germline SNPs on patient outcome post-diagnosis is less well understood. Here, we have shown that cell type-restricted eQTLs can be identified by modelling tumor purity as part of the standard eQTL analysis. We identified examples of germline SNPs near ieGenes that are associated with progression-free survival (LPP and EZH2), demonstrating the potential importance of germline SNPs acting through gene expression in cancer patient outcome post diagnosis. These results point to the importance of both considering all contributors to gene expression variance in cancer including germ line polymorphisms as well as the potential clinical importance of germline variation post cancer diagnosis.

## Supplementary Information

**Additional file 1:**

**Additional file 2:** 

**Additional file 3:** 

**Additional file 4:** 

**Additional file 5:** 

**Additional file 6:** 

**Additional file 7:** 

**Additional file 8:** 

## Data Availability

All data used in this study are available to the public. Somatic alteration data, gene expression data and clinical data from the TCGA used during the current study are available in the Genomics Data Commons repository (https://gdc.cancer.gov/). Germline data are hosted at dbGaP (https://dbgap.ncbi.nlm.nih.gov) and are available to the public following approval.

## Abbreviations

Eqtl: Expression quantitative trait loci
CIN: Chromosomal instability
ER + /ER: Estrogen-receptor positive/negative
FDR: False discovery rate
GDC: Genomics Data Commons
GO: Gene Ontology
GTEx Project: Genotype Expression Project
PCA: Principal component analysis
SNP: Single nucleotide polymorphism
somQTL: Somatic quantitative trait loci
TCGA: The Cancer Genome Atlas
TMB: Tumor mutation burden
