# Supplementary figures and images for: Contribution and clinical relevance of germline variation to the cancer transcriptome
Source: BMC Cancer. 2022 Jun 20;22:675. doi: 10.1186/s12885-022-09757-0 (PMC9208227; doi:10.1186/s12885-022-09757-0)

A

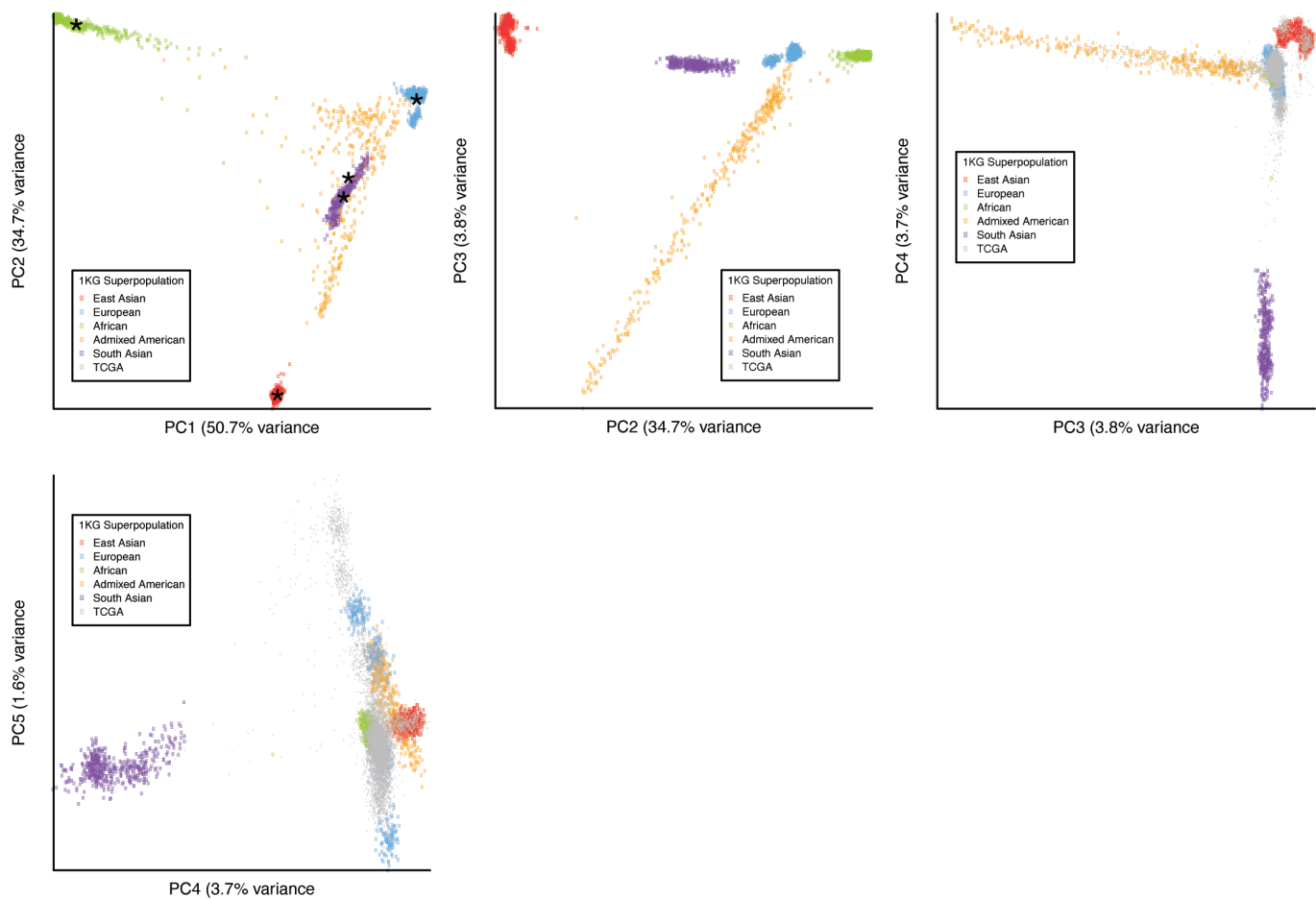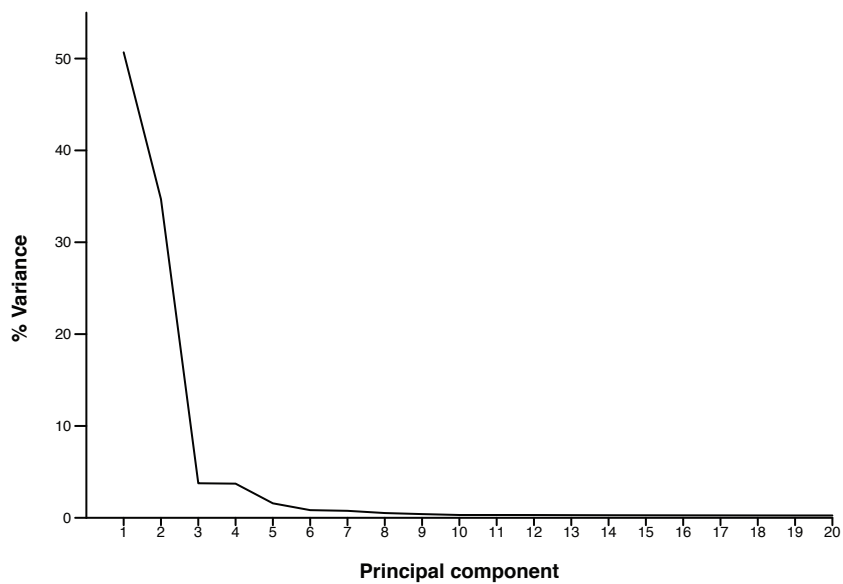

Supplement: Supplementary file 1 — Additional file 1: [file 12885_2022_9757_MOESM1_ESM.pdf]

**A**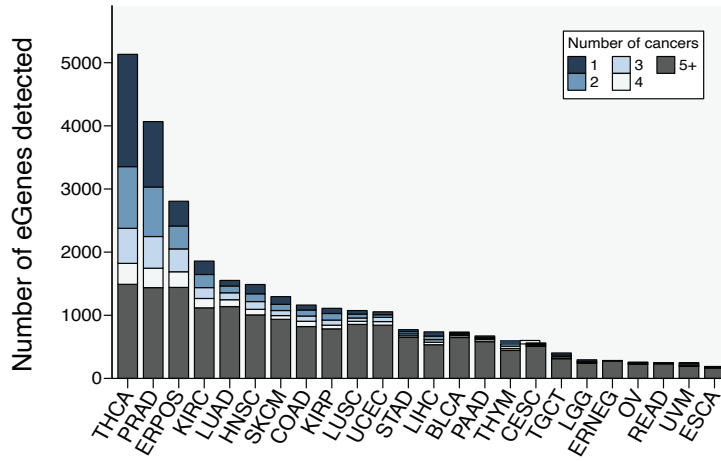**B**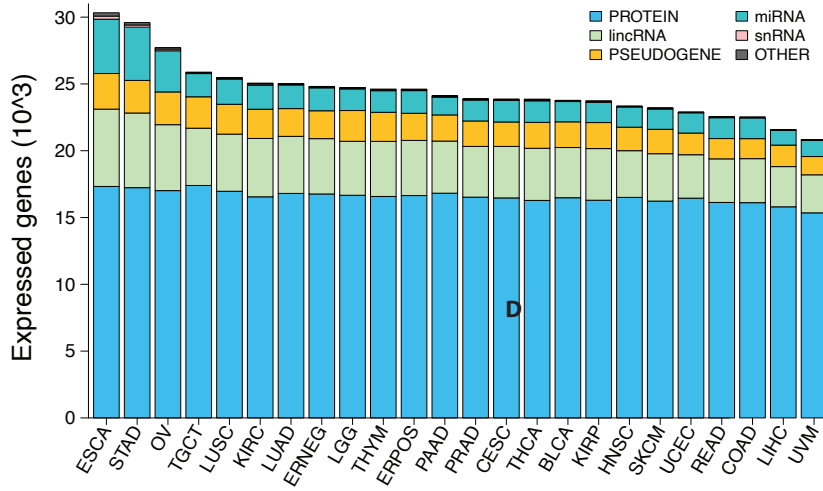

Supplement: Supplementary file 2 — Additional file 2: [file 12885_2022_9757_MOESM2_ESM.pdf]

**R = 0.83, p = 4.7e-07**

eGene/expressed genes

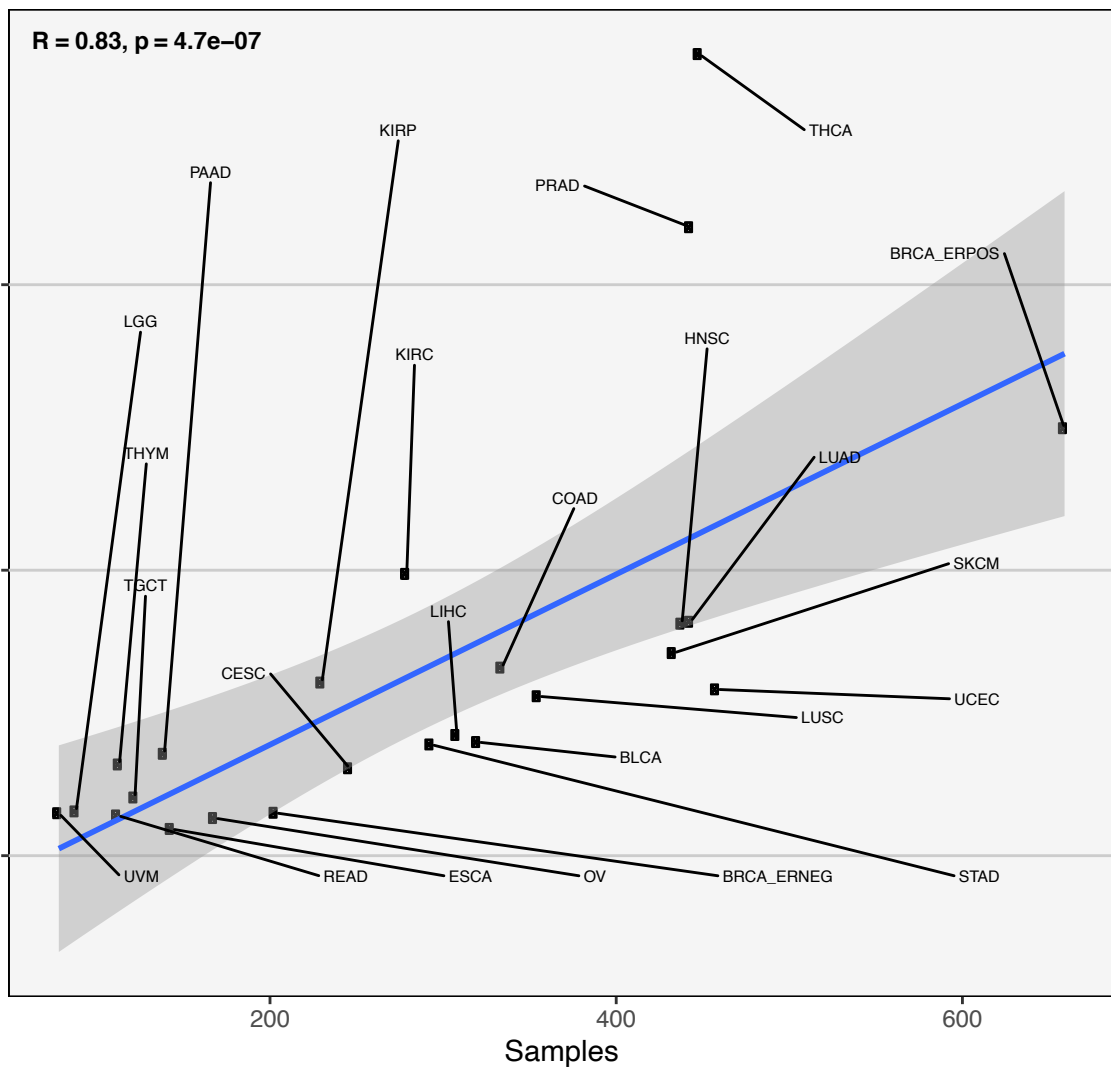

Supplement: Supplementary file 3 — Additional file 3: [file 12885_2022_9757_MOESM3_ESM.pdf]

**A**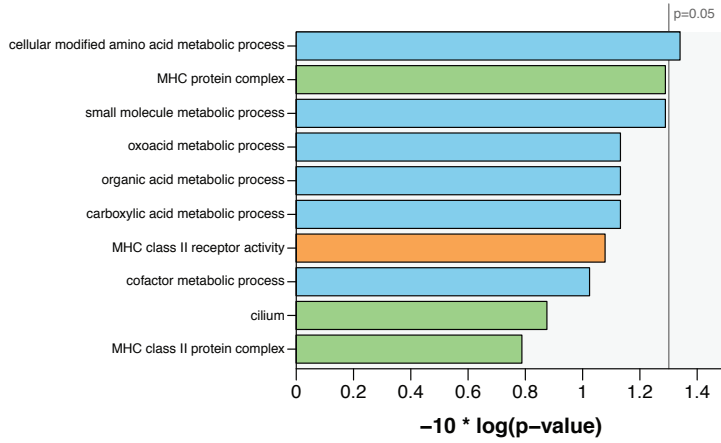**B**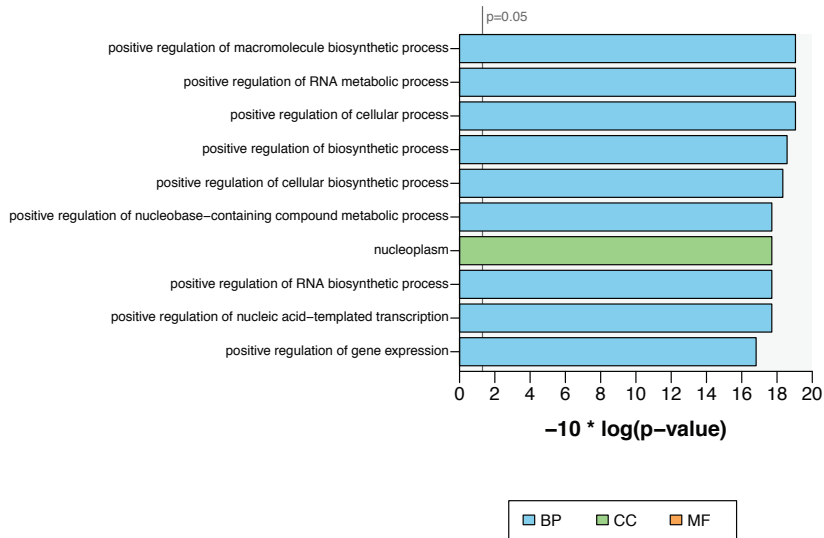

Supplement: Supplementary file 4 — Additional file 4: [file 12885_2022_9757_MOESM4_ESM.pdf]

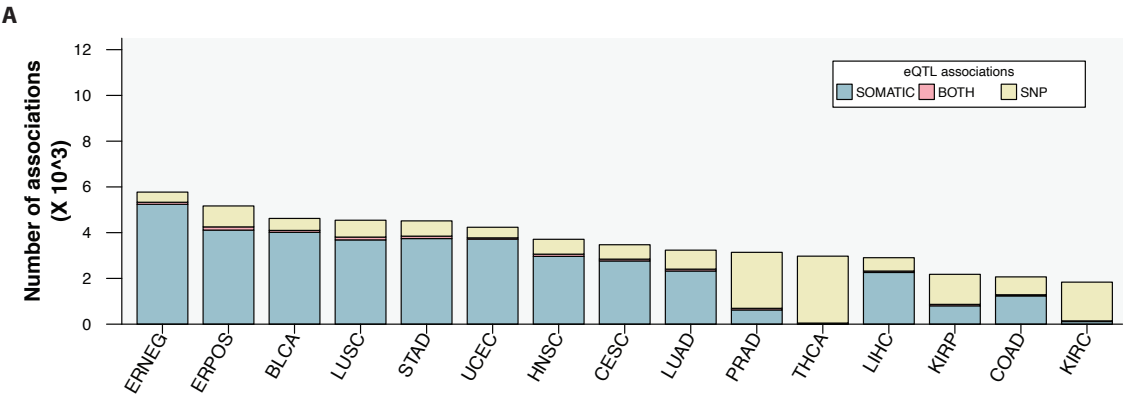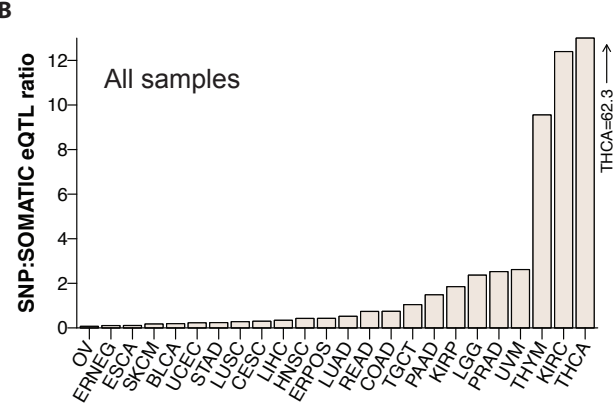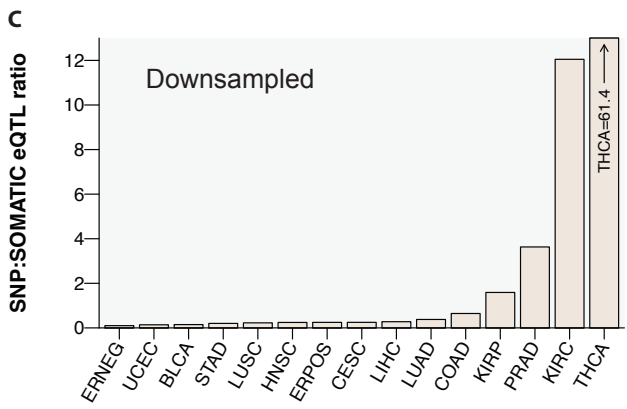

Supplement: Supplementary file 5 — Additional file 5: [file 12885_2022_9757_MOESM5_ESM.pdf]

**A**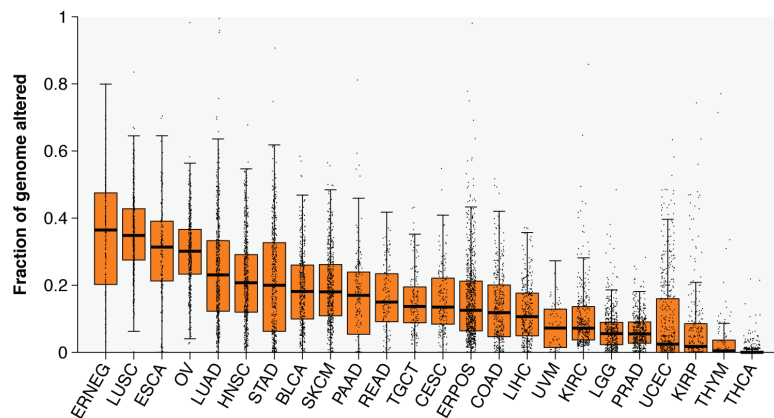**B**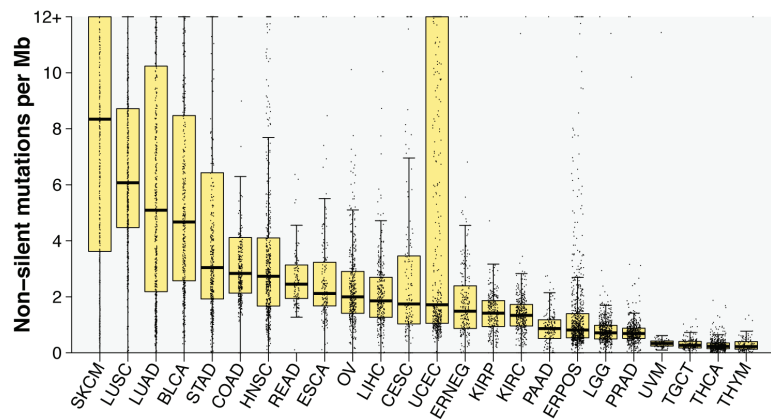**C**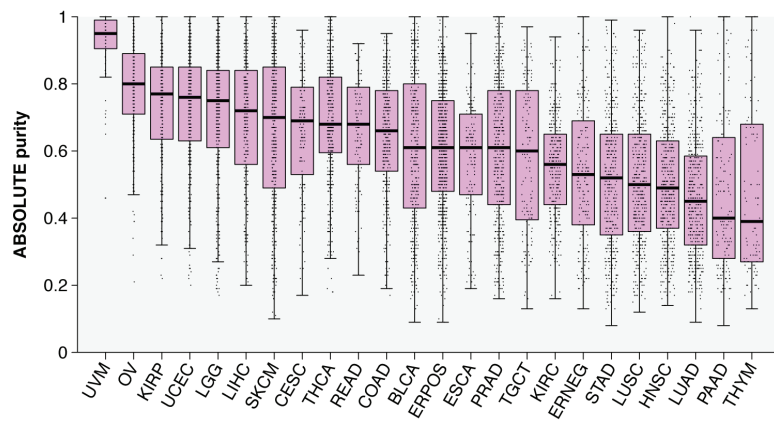

Supplement: Supplementary file 6 — Additional file 6: [file 12885_2022_9757_MOESM6_ESM.pdf]

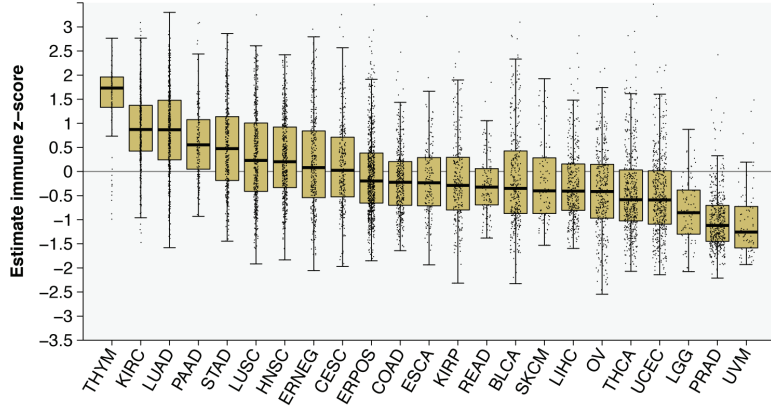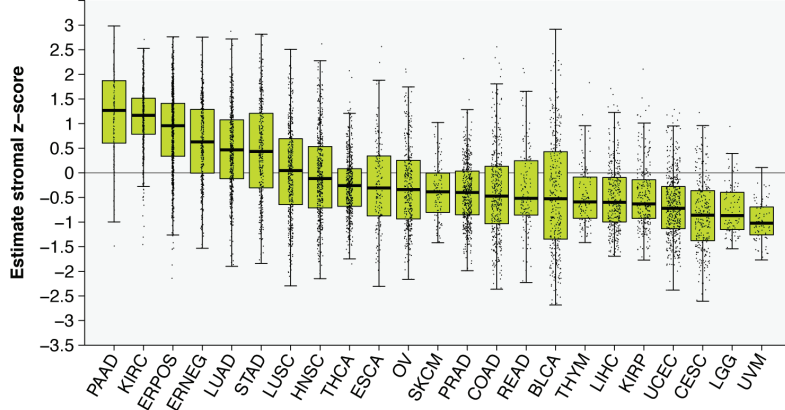

Supplement: Supplementary file 7 — Additional file 7: [file 12885_2022_9757_MOESM7_ESM.pdf]

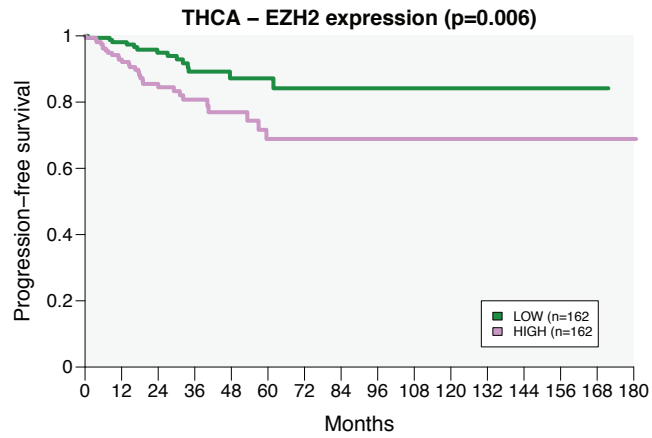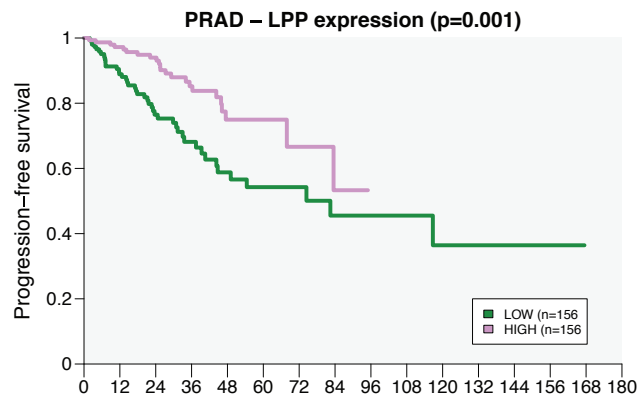

Supplement: Supplementary file 8 — Additional file 8: [file 12885_2022_9757_MOESM8_ESM.pdf]
